# CME ACTIVITY: β-Herpesviruses in Febrile Children with Cancer

**DOI:** 10.3201/eid1404.0706511

**Published:** 2008-04

Medscape, LLC is pleased to provide online continuing medical education (CME) for this journal article, allowing clinicians the opportunity to earn CME credit. Medscape, LLC is accredited by the Accreditation Council for Continuing Medical Education (ACCME) to provide CME for physicians. Medscape, LLC designates this educational activity for a maximum of 1.0 *AMA PRA Category 1 Credits*™. Physicians should only claim credit commensurate with the extent of their participation in the activity. All other clinicians completing this activity will be issued a certificate of participation. To participate in this journal CME activity: (1) review the learning objectives and author disclosures; (2) study the education content; (3) take the post-test and/or complete the evaluation at **http://www.medscape.com/cme/eid**; (4) view/print certificate.

## Learning Objectives

Upon completion of this activity, participants will be able to:

- Identify common infections associated with β-herpesviruses
- Specify β-herpesviruses isolated from children in the current study
- Describe clinical characteristics of β-herpesvirus infections in the current study
- List factors associated with higher rates of infection with human herpesvirus in the current study

## Editor

**D. Peter Drotman, MD**, Editor-in-Chief, Emerging Infectious Diseases. *Disclosure: D. Peter Drotman, MD, has disclosed no relevant financial relationships.*

## CME Author

**Charles P. Vega, MD**, Associate Professor; Residency Director, Department of Family Medicine, University of California, Irvine. *Disclosure: Charles P. Vega, MD, has disclosed that he has served as an advisor or consultant to Novartis, Inc*

## Authors

**Stephanie Yee-Guardino, DO**, Section of Pediatric Infectious Diseases, Cleveland Clinic Children’s Hospital, Cleveland, Ohio. *Disclosure: Stephanie Yee-Guardino, DO, has disclosed no relevant financial relationships.*

**Kate Gowans, MD**, Department of Pediatric Hematology and Oncology, Cleveland Clinic Children’s Hospital, Cleveland, Ohio.* Disclosure: Kate Gowans, MD, has disclosed no relevant financial relationships.*

**Belinda Yen-Lieberman, PhD**, Laboratory Medicine, Lerner Research Institute, Cleveland Clinic, Cleveland, Ohio. *Disclosure: Belinda Yen-Lieberman, PhD, has disclosed that she has received reagents at no charge from ARTUS Diagnostics.*

**Pamela Berk, BS**, Department of Molecular Genetics, Lerner Research Institute, Cleveland Clinic, Cleveland, Ohio. *Disclosure: Pamela Berk, BS, has disclosed no relevant financial relationships.*

**Debra Kohn, BS, MT**, Laboratory Medicine, Lerner Research Institute, Cleveland Clinic, Cleveland, Ohio. *Disclosure: Debra Kohn, BS, MT, has disclosed no relevant financial relationships.*

**Fu-Zhang Wang, PhD**, Department of Molecular Genetics, Lerner Research, Institute Cleveland Clinic, Cleveland, Ohio. *Disclosure: Fu-Zhang Wang, PhD, has disclosed no relevant financial relationships.*

**Lara Danziger-Isakov, MD, MPH**, Section of Pediatric Infectious Diseases, Cleveland Clinic Children’s Hospital, Cleveland, Ohio; Departments of Infectious Diseases, Lerner Research Institute, Cleveland Clinic, Cleveland, Ohio. *Disclosure: Lara Danziger-Isakov, MD, MPH, has disclosed no relevant financial relationships.*

**Camille Sabella, MD**, Section of Pediatric Infectious Diseases, Cleveland Clinic Children’s Hospital, Cleveland, Ohio; Departments of Infectious Diseases, Lerner Research Institute, Cleveland Clinic, Cleveland, Ohio. *Disclosure: Camille Sabella, MD, has disclosed that she has received grants from Merck, Inc. and Sanofi Pasteur.*

**Sarah Worley, MS**, Quantitative Health Sciences, Lerner Research Institute, Cleveland Clinic, Cleveland, Ohio. Disclosure: Sarah Worley, MS, has disclosed no relevant financial relationships.

**Philip E. Pellett, PhD**, Departments of Infectious Diseases, Department of Molecular Genetics, Lerner Research Institute, Cleveland Clinic, Cleveland, Ohio. Disclosure: Philip E. Pellett, PhD, has disclosed that he has received royalties from Chemicon International.

**Johanna Goldfarb, MD**, Section of Pediatric Infectious Diseases, Children's Hospital, Cleveland, Ohio; Departments of Infectious Diseases, Lerner Research Institute, Cleveland Clinic, Cleveland, Ohio. Disclosure: Johanna Goldfarb, MD, has disclosed no relevant financial relationships.*.*

## Earning CME Credit

To obtain credit, you should first read the journal article. After reading the article, you should be able to answer the following, related, multiple-choice questions. To complete the questions and earn continuing medical education (CME) credit, please go to **http://www.medscape.com/cme/eid**. Credit cannot be obtained for tests completed on paper, although you may use the worksheet below to keep a record of your answers. You must be a registered user on Medscape.com. If you are not registered on Medscape.com, please click on the New Users: Free Registration link on the left hand side of the website to register. Only one answer is correct for each question. Once you successfully answer all post-test questions you will be able to view and/or print your certificate. For questions regarding the content of this activity, contact the accredited provider, CME@medscape.net. For technical assistance, contact CME@webmd.net. American Medical Association’s Physician’s Recognition Award (AMA PRA) credits are accepted in the US as evidence of participation in CME activities. For further information on this award, please refer to http://www.ama-assn.org/ama/pub/category/2922.html. The AMA has determined that physicians not licensed in the US who participate in this CME activity are eligible for *AMA PRA Category 1 Credits*™. Through agreements that the AMA has made with agencies in some countries, AMA PRA credit is acceptable as evidence of participation in CME activities. If you are not licensed in the US and want to obtain an AMA PRA CME credit, please complete the questions online, print the certificate and present it to your national medical association.

## CME Questions

1. Which of the following statements about β-herpesviruses is *most* accurate?
  A. Infection is not common until late adolescence
  B. Infection with cytomegalovirus (CMV) promotes symptoms similar to mononucleosis
  C. Human herpesvirus (HHV)-6B is the cause of fifth disease
  D. HHV-7 is the virus responsible for most cases of roseola
2. Which of the following β-herpesviruses were detected in the patient cohort of the current study?
  A. HHV-6B and HHV-6A
  B. CMV and HHV-7
  C. HHV-6B and CMV
  D. HHV-6A and HHV-7
3. Which of the following statements about infection data in the current study is *most* accurate?
  A. The etiology of most patients’ fever was discovered during hospitalization
  B. Infection with β-herpesviruses occurred at a higher frequency among children with cancer compared with those with solid organ transplant
  C. Fever was generally higher among children infected with β-herpesviruses
  D. HHV-6B infection was likely a reactivation of previous infection among cancer patients
4. Which cancer factors promoted infection with HHV-6B in the current study?
  A. Solid organ tumor and over 6 months since the initiation of immune suppression
  B. Solid organ tumor and less than 6 months since the initiation of immune suppression
  C. Leukemia and less than 6 months since the initiation of immune suppression
  D. Leukemia and more than 6 months since the initiation of immune suppression

**Table:** Activity Evaluation

**1. The activity supported the learning objectives.**				
Strongly Disagree				Strongly Agree
1	2	3	4	5
**2. The material was organized clearly for learning to occur.**				
Strongly Disagree				Strongly Agree
1	2	3	4	5
**3. The content learned from this activity will impact my practice.**				
Strongly Disagree				Strongly Agree
1	2	3	4	5
**4. The activity was presented objectively and free of commercial bias.**				
Strongly Disagree				Strongly Agree
1	2	3	4	5

